# *Ziziphora clinopodioides* Essential Oil and Nisin as Potential Antimicrobial Agents against* Escherichia coli* O157:H7 in Doogh (Iranian Yoghurt Drink)

**DOI:** 10.1155/2015/176024

**Published:** 2015-12-13

**Authors:** Yasser Shahbazi

**Affiliations:** Department of Food Hygiene and Quality Control, Faculty of Veterinary Medicine, Razi University, Kermanshah 14199-63111, Iran

## Abstract

The aim of the present study was to evaluate the effects of* Ziziphora clinopodioides* essential oil (0.1 and 0.2%) and nisin (250 and 500 IU/mL) separately and in combination on survival of* Escherichia coli* O157:H7 inoculated in Doogh (Iranian yoghurt drink) during storage under refrigerated temperature (4 ± 1°C) for 9 days. Viability of* Lactobacillus casei* at different concentrations of* Z. clinopodioides* essential oil (0.1 and 0.2%) in Doogh was also examined. The major components were carvacrol (64.22%), thymol (19.22%), *γ*-terpinene (4.63%), and *p*-cymene (4.86%). There was no significant difference (*p* > 0.05) between samples treated with nisin and those of untreated samples. Samples treated with both concentrations of the essential oil (0.1 and 0.2%) showed populations of* E. coli* O157:H7 significantly (*p* < 0.05) lower than those of untreated samples. The essential oil of* Z. clinopodioides* in combination with nisin had a potential synergistic effect against* E. coli* O157:H7 in Doogh samples after 5 days. The count of* L. casei* was not inhibited by different concentrations of the* Z. clinopodioides* essential oil. It is concluded that the leaf essential oil of* Z. clinopodioides* in combination with nisin can be applied as alternative antimicrobial agents in Doogh to inhibit the growth of* E. coli* O157:H7.

## 1. Introduction

In recent years, diseases caused by food-borne pathogens such as* Escherichia coli* O157:H7 are serious concerns for economic and public health.* E. coli* O157:H7 is the most important member of a group of pathogenic* E. coli* strains that variously are enterohaemorrhagic, verocytotoxin, or Shiga-toxin-producing organisms [1, 2]. The most clinical diseases caused by this bacterium includes diarrhoea, haemorrhagic colitis, and the haemolytic uraemic syndrome (HUS) which is the leading cause of acute renal failure in children [3–5]. It was recognized as the most heat and acid resistant pathogen in foods and numerous researches reported that it can survive in acidic products such as dairy beverages and fruit juices [2, 6]. The most common foods as potential sources of this microorganism include undercooked ground beef, milk and dairy products, and juices [7]. Doogh is the most commonly consumed Iranian yoghurt drink with a long history of manufacturing. Traditionally, it is prepared by full-fat yoghurt, water, salt, and sweet-smelling herbs mixing in special leather bag (called Mashk in Persian language). Salt at a maximum level of 1% and herbs such as zizifore, mint, oregano, and thyme are added to impart flavour [8, 9]. Recently, several Iranian researchers performed studies on the pathogens carried via Doogh; they have illustrated that* E. coli* O157:H7, amongst other pathogenic bacteria such as* Salmonella typhimurium* and* Staphylococcus aureus*, is highly prevalent [10, 11].

A lot of study is being done to make new natural preservation treatments in an attempt to control of food-borne pathogens while keeping a high organoleptic and nutritional quality of the food products [12]. In this way, the combination of antimicrobial compounds such as essential oils with bacteriocins can provide an enhanced antimicrobial effect, resulting in fewer undesirable effects [6]. In relation to essential oils, numerous studies have been demonstrated to have antimicrobial activity against food-borne pathogens [1, 2, 13–15].* Ziziphora clinopodioides* is one of the most commonly consumed medicinal edible plants, belonging to the Lamiaceae family, that widely distributed in Asia and Europe especially Turkey and west of Iran (from flora of Iran especially in Ilam, Kurdistan, Kermanshah and Lorestan provinces). The fresh and dried plant and its essential oil are widely used as medicinal for diarrhoea, intestinal gas, nausea, and vomiting. This plant is extensively used as flavour ingredient in a wide variety of foods in Iran [16–18]. The major constituents of the essential oil of this plant that has medicinal properties include pulegone, 1,8-cineole, thymol, carvacrol,* p*-cymene, and limonene [18]. To establish the efficacy of natural antimicrobial agents as food preservatives, they must be examined separately and in combination with other preservative agents such as nisin in various food model systems to determine whether there are synergistic effects and multiple hurdles can be devised [19, 20]. Nisin is the only bacteriocin that has been permitted to be applied in the foods in particular milk and dairy products in over fifty countries. It is a peptide composed of 34 amino acid residues, with a molecular mass of 3.5 kDa, and is classified as a class-Ia bacteriocin or lantibiotic. This antibacterial peptide is produced by certain strains of* Lactococcus lactis* subsp.* lactis* and its importance is related to its wide spectrum of effect on growth of Gram-negative and Gram-positive bacteria [21].

To date, antibacterial effects of various essential oils of medicinal plants, separately and in combination with other natural antibacterial agents such as bacteriocin, has been studied in different food model systems throughout the world [1, 2, 5, 7, 14, 15]. However, to the best of our knowledge, there has been no published detailed information about the antibacterial activity of* Z. clinopodioides* essential oil in foods, in particular Doogh. Hence, the aim of the present study was to evaluate the effects of* Z. clinopodioides* essential oil and nisin separately and in combination on survival of* E. coli* O157:H7 inoculated in Doogh during storage under refrigerated temperature (4 ± 1°C) for 9 days. Viability of* Lactobacillus casei* at different concentrations of* Z. clinopodioides* essential oil (0.1 and 0.2%) in Doogh was also examined.

## 2. Materials and Methods 

### 2.1. Plant Material

The leaf part of* Z. clinopodioides* plant was collected during March–July 2014 from Gilan-e Gharb area (Kermanshah province, Western Iran) at a latitude, longitude, and altitude of 3,776,583 Universal Transverse Mercator (UTM), 585,86 UTM, and 833 m above sea level, respectively. The plant was authenticated by Dr. Seyed Mohammad Masoumi, Faculty of Agriculture, Razi University, Kermanshah, Iran. Vouchers specimen (number 6816) of the plant was deposited in the herbarium of the Research Center of Natural Resources of Tehran, Iran.

### 2.2. Isolation of Essential Oil

The one hundred grams (100 g) of dried leaves were submitted to hydrodistillation for 3.5 h using an all-glass Clevenger apparatus as recommended by the European Pharmacopoeia [22]. Then, it was heated by heating mantle until the water boiled. After collecting the crude essential oil using a micropipette from above the distillate without adding any solvent in a sealed bottle, it was dried over anhydrous sodium sulfate (Na_2_SO_4_) (Merck, Darmstadt, Germany) until the last traces of water removing and, after filtration, kept in a dark sealed glass bottle at 4 ± 1°C until GC-MS analyses and further use.

### 2.3. Gas Chromatography-Mass Spectrometry (GC-MS) Analysis of Essential Oil

GC-MS analysis of* Z. clinopodioides* essential oil was carried out on a gas chromatography (Thermo Quest 2000, UK) coupled with mass spectrometer detector (Thermo Quest Finnigan, UK) (GC-MS) equipped with HP-5MS 5% phenyl methylsiloxane capillary column (30.00 m length × 0.25 mm ID, 0.25 *μ*m film thickness). The electron impact mode system (ionization energy: 70 eV) was used over a scan range of 30–550 amu (atomic mass unit) for the ionization and separation of the compounds. Helium was a carrier gas with a constant flow rate of 1.2 mL/min. The temperature of mass transfer line and injector was set at 300°C and 290°C, respectively. The oven temperature was programmed from 50°C (hold 3 min) to 265°C at 2.5°C/min, then kept isothermal for 20 min, and finally raised to 265°C at 6°C/min. 1 *μ*L of the essential oil was injected in the split mode with a split ratio of 20 : 1. Analysis of the essential oil also was done by gas chromatography (Thermo Quest Finnigan, UK). The capillary column and temperature condition was similar to gas chromatography coupled with a mass spectrometer as described above.

### 2.4. Identification of Chemical Compounds

The chemical compounds of the essential oil were identified on the basis of GC-MS retention time on fused silica capillary column and by comparison between their retention indices (RIs) with retention indices of published data, Standard Mass Spectral Fragmentation Pattern (Wiley/NBS Pak v.7, 2003) and the National Institute of Standards and Technology (NIST; v.2.0, 2005). The GC peak area normalization of the three injections was expressed as mean percentage of individual essential oil composition.

### 2.5. Preparation of Nisin

Nisin with a label activity of 10^4^ International Units (IU/g) was supplied by Sigma-Aldrich Company, UK. Appropriate amount of nisin was suspended in 0.02 M HCl, centrifuged at 1500 ×g for 20 min; the supernatants were sterilized by 0.22 *μ*m filter (Sigma-Aldrich, UK) and kept at −20°C until use [23]. To obtain the desired concentration of nisin, the stock solution was thawed at 25°C and diluted appropriately in sterile water, yielding final concentrations of 250 or 500 IU/mL nisin.

### 2.6. Test Microorganisms

*E. coli* O157:H7 (ATCC 10536) and* L. casei* (ATCC 393) as lyophilized cultures were purchased from the culture collection of the Iranian Research Organization for Science and Technology (IROST), Tehran, Iran. Before the test, the bacterial strains were routinely grown on Brain Heart Infusion broth (BHI; Merck, Darmstadt, Germany) medium at 37°C for 18 h and enumerated by Brain Heart Infusion agar (BHI; Merck, Darmstadt, Germany) medium in triplicate at the same incubation condition. The optical densities of the 18 h old cultures of the strains were determined spectrophotometrically at 600 nm (for* E. coli* O157:H7: 1 × 10^5^ CFU/mL and for* L. casei*: 3.5 × 10^8^ CFU/mL) using sterile BHI broth. Bacterial cells were assessed by using plating on BHI agar and counting viable cells after incubation for 24 h at 37°C in triplicate.

### 2.7. Determination of the Minimum Inhibitory Concentration (MIC) of* Z. clinopodioides* Essential Oil and Nisin

In order to determine the minimum inhibitory concentration (MIC) of* Z. clinopodioides* essential oil and nisin, a broth microdilution test was used. For this purpose, 5% (v/v) dimethyl sulfoxide (DMSO) (Merck, Darmstadt, Germany) as an emulsifier and 0.05% (w/v) agar-agar (Merck, Darmstadt, Germany) as a stabilizer of the essential oil were added to BHI broth medium. After autoclaving of media, different concentrations of the essential oil (0.0125, 0.025, 0.05, 0.1, 0.25, 0.5, 1, 1.5, and 2%) and nisin (3.75, 7.5, 15, 30, 60, 125, 250, 500, and 1000 IU/mL) were set up using 96-well sterile microdilution plates with U-bottom wells. Then, 180 *μ*L BHI broth containing different concentrations of the essential oil and nisin and 20 *μ*L of the final bacterial inoculum (1 × 10^6^ CFU/mL) were added into each well. As a positive control, the same amount of BHI broth containing DMSO and bacterium inoculum without essential oil and nisin was added into well. Moreover, in each experiment, negative controls, BHI broth containing DMSO and essential oil and nisin, were considered. Then, the content of plates were shaken for 30 s and incubated at 37°C for 24 h. The MIC was described as the lowest concentration of essential oil and nisin that prevent the growth of the microorganism. For* E. coli* O157:H7, the MICs of the essential oil and nisin were 0.05% and 125 IU/mL, respectively. Hence, twofold the minimum inhibitory concentration (MIC) values were considered for evaluating of the antimicrobial activities of the essential oil and nisin in Doogh samples.

### 2.8. Preparation of Doogh

Full-fat yoghurt was purchased from a local store of Kermanshah city, west of Iran. Before the test, the total solid (TS), pH, total lipid, total sugar, protein, and ash were measured. Then, Doogh sample was prepared by addition of yoghurt (3.5 g/100 g total lipid, 3.52 g/100 g protein, 0.8 g/100 g ash, 14.3 g/100 g total solid, and 5.32 g/100 g total sugar) and water at the ratio of 1 : 1, followed by thorough mixing for 30 s. After this step, NaCl was added with Doogh sample at the ratio of 1 g/100 mL and sample gently stomached for 30 s at room temperature. In the present study, the Doogh samples divided into three groups (group 1: without inoculated bacteria; group 2: inoculated with* L. casei;* and group 3: inoculated with* E. coli* O157:H7). In the group dosed with bacteria, 100 mL of Doogh samples was poured into Erlenmeyer flask, inoculated with 5 log CFU/mL and 8.5 log CFU/mL of* E. coli* O157:H7 and* L. casei*, respectively. Then, the samples were shaken for 2 min to ensure uniform distribution of the bacteria. After homogenization,* Z. clinopodioides* essential oil (0.1 and 0.2%) and nisin (250, and 500 IU/mL), separately and in combination, were added into the samples. The same procedure was done in group 1, except inoculation of the bacteria. The samples were stored at refrigerated temperature (4 ± 1°C) and used for further analysis at 0, 1, 3, 5, 7, and 9 days. All experiments were conducted in independent triplicate.

### 2.9. Microbiological Analysis

For microbiological analysis, sampling was done on days 0, 1, 3, 5, 7, and 9. At each sampling day, two samples were analyzed. Each time, 10 mL of Doogh samples was transferred aseptically to a stomacher bag and diluted with 90 mL of 0.1 g/100 mL sterile buffered peptone water (Merck, Darmstadt, Germany). Then, sample was homogenized in a stomacher for 30 s, subsequently diluted in 0.1 g/100 mL sterile buffered peptone water, and then surface-plated onto Eosin methylene blue agar (for* E. coli* O157:H7) and Reconstituted Clostridial Agar with bromocresol green and vancomycin (RCABV) (for* L. casei*). Plates were incubated at 37 ± 2°C for 24–48 h. Results were expressed as log CFU/mL.

### 2.10. Sensory Evaluation

The sensory effects of adding of* Z. clinopodioides* essential oil and nisin to Doogh samples were evaluated using an acceptance test. A panel of seven judges experienced in dairy product evaluation was used for sensory analysis. Panelists were asked to evaluate odour and flavour of samples. Acceptability of samples was estimated using an acceptability scale ranging from 10 to 1 with 10 corresponding to the most liked sample and 1 corresponding to the least liked sample.

### 2.11. Statistical Analysis

SPSS 16.0 for Windows (SPSS, Chicago, IL, USA) software package was used for data analyses. Mean and standard deviations of each experiment were calculated and then were subjected to analysis of variance. Tukey's test at 95% confidence interval was used to determine mean differences among the treatments.

## 3. Results and Discussion 

### 3.1. Chemical Composition of* Z. clinopodioides* Essential Oil

The hydrodistillation of 100 g of the fine-powdered plant leaf yielded 0.65% v/w greenish oil with a distinct smell. The results of the oil yield were in accordance with Behravan et al. (2007) reporting the yield of oils to be 0.75% [18]. The essential oil sample was analyzed by GC-MS and the compounds were identified on the basis of their retention indices values and by comparison of their mass spectra with those reported in the literatures. The GC-MS analysis of the oil showed the presence of 24 compounds, accounting for 99.65% of the oil. Its percentage compositions are shown in Table 1. Among them, the amount of the oxygenated monoterpene fraction was 86.1% of the oil while the monoterpene hydrocarbons fraction was 11.97%. The sesquiterpene hydrocarbons fraction was 1.07% and the oxygenated sesquiterpenoid fraction was 0.43% in the oil. The major components were phenolic compounds including carvacrol (64.22%), thymol (19.22%), *γ*-terpinene (4.63%), and* p-*cymene (4.86%). The results of chemical composition of the present study are in agreement with Aghajani et al. (2008) [16] who reported that carvacrol (8.7%) and thymol (53.6%) were the main constituents of the essential oil of* Z. clinopodioides* plant collected from Lorestan province, west of Iran. In contrast with our results, Behravan et al. (2007) [18] and Ozturk and Ercisli (2007) [24] reported that pulegone, terpineol, and 1,8-cineole were the most abundant compounds of* Z. clinopodioides* essential oil harvested from Iran and Turkey, respectively. These observed differences in the chemical compositions of* Z. clinopodioides* could be related to occurrence of chemotypes, geographical locations, season at the time of collection, stage of development, culture climate, and other culture conditions, which may affect biological activities [13, 14]. It is well known that the greater the amount of phenolic compounds such as carvacrol and thymol the greater the antimicrobial activity showed by the essential oil. This may be due to the acidic nature of their hydroxyl group and involvement in the formation of hydrogen bonds [25].

### 3.2. Survival of* E. coli* O157:H7 and* L. casei* in Doogh Stored at 4°C

The results of growth inhibition of* E. coli* O157:H7 in Doogh by different concentrations of* Z. clinopodioides* and nisin are shown in Table 2. The initial population of the microorganism (5 log CFU/mL) decreased to 2.15 log CFU/mL in 5 days of storage in untreated samples. Based on our results, there was no significant difference (*p* > 0.05) between samples treated with nisin and untreated samples. The hydrophobic lipopolysaccharide present in the outer layer of Gram-negative bacteria such as* E. coli* O157:H7 might be responsible for their more enhanced resistance to nisin [26]. Moreover, the populations of* E. coli* O157:H7 in samples treated with different concentrations of the essential oil were kept below 1 log CFU/mL on day 5 of storage. Indeed, samples treated with both concentrations of the essential oil (0.1 and 0.2%) showed populations of* E. coli* O157:H7 significantly (*p* < 0.05) lower than those of untreated samples. The numbers of surviving bacteria following exposure to the essential oil did not significantly vary (*p* > 0.05) between two concentrations. According to results of the present study, it was found that leaf essential oil* Z. clinopodioides* (0.1 and 0.2%) in combination with different concentrations of nisin (250 and 500 IU/mL) had a potential synergistic effect against* E. coli* O157:H7 in Doogh sample after 5 days. A strong antibacterial effect was observed when Doogh samples were treated with 0.1 and 0.2% essential oil in combination with different concentrations of nisin. In these groups of treated samples, the growth of bacterium completely inhibited and no cell count number were observed on day 5 of storage. Moreover, the results obtained for combination concentrations of the essential oil and nisin demonstrated that the combination of the essential oil at 0.2% and nisin at 500 IU/mL significantly (*p* < 0.05) decreased the population of pathogen when compared with other treated sample groups. The antibacterial effect of the* Z. clinopodioides* essential oil and nisin, separately and in combination, against* E. coli* O157:H7 in Doogh had not yet been reported.* In vitro* studies showed that the combination of the essential oil and nisin had a greater efficacy than the essential oil or nisin separately against food-borne pathogens especially* E. coli* O157:H7 [27–29].

**Figure 1:**
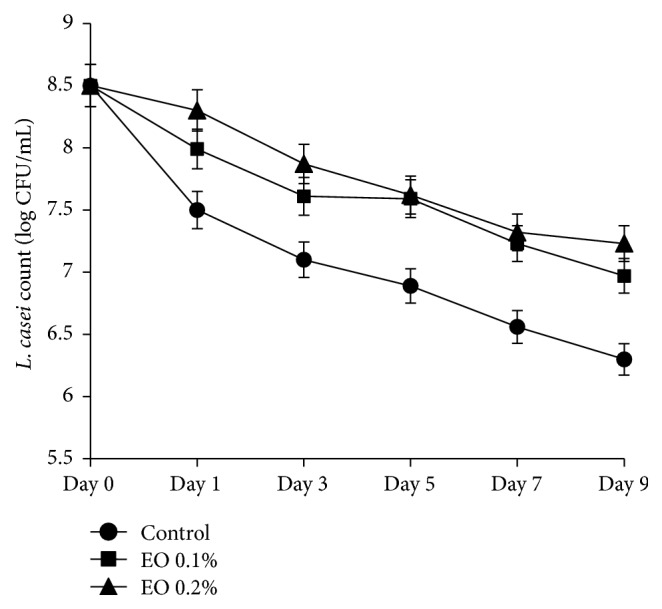
Viability of* L. casei* (log CFU/mL) at different concentrations of* Z. clinopodioides* essential oil (0.1 and 0.2%) in Doogh samples during storage period.

According to results of this work, following storage of time a significant reduction of the count of* E. coli* was observed. Several factors may contribute to reduction of this pathogen during storage such as the presence of lactic acid bacteria. The progressive production of some compounds such as hydrogen peroxide and volatile compounds by lactic acid bacteria is well documented. A number of studies have shown the inhibitory effects of these compounds against food-borne pathogens [30, 31]. Moreover, earlier studies have shown that most food-borne pathogens are susceptible to the lethal of low pH [32–34]. Indeed, acidic property (pH) of Doogh would be a main key factor influencing the decrease of survival and growth of bacterial pathogens such as* E. coli* O157:H7. These results are similar to that reported other authors [33, 34]. As shown in Figure 1, the count of* L. casei* was not inhibited by different concentrations of the* Z. clinopodioides* essential oil. Previous study showed that some of the essential oils such as thyme had a stimulatory effect on lactic acid bacteria by enhancing their growth and acid production [35]. Based on our findings, Doogh can be an excellent food to carry relevant probiotic bacteria while adding the* Z. clinopodioides* essential oil.

### 3.3. Sensory Properties

Acceptability scores (odour and flavour properties) of Doogh samples for all different treatments are shown in Table 3. There were significant differences (*p* < 0.05) in the odour and flavour of treated samples as compared with the untreated control. It should be noted that* Z. clinopodioides* (at 0.1% concentration) flavour was very delicate and it did not hamper sensory evaluation of samples. Likewise, nisin at 250 and 500 IU/mL did not affect sample sensory properties. Our results are in agreement with previous study [35].

## 4. Conclusions

In conclusion, our findings indicated that synergistic combinations of* Z. clinopodioides* essential oil with nisin have significant inhibitory effects against* E. coli* O157:H7 in Doogh samples. Hence, it is concluded that the leaf essential oil of* Z. clinopodioides* in combination with nisin can be applied as an alternative antimicrobial agent in Doogh to inhibit the growth of* E. coli* O157:H7.

## Figures and Tables

**Table 1:** Essential oil composition of *Z*. *clinopodioides* identified by GC-MS.

Number	Compound name	Composition%	Retention time (min)	Kovats index
1	*α*-Thujene	0.26	11.33	927
2	*α*-Pinene	0.27	11.71	934
3	Camphene	0.13	12.61	952
4	*β*-Pinene	0.06	14.06	981
5	1-Octen-3-ol	0.08	14.32	986
6	Myrcene	0.51	14.62	992
7	*α*-Phellandrene	0.13	15.58	1010
8	*α*-Terpinene	0.79	16.11	1021
9	*p*-Cymene	4.86	16.62	1030
10	Limonene	0.1	16.77	1033
11	*β*-Phellandrene	0.11	16.89	1036
12	*γ*-Terpinene	4.63	18.31	1063
13	*cis*-Sabinene hydrate	0.07	19.02	1077
14	Terpinolene	0.08	19.69	1089
15	Linalool	0.13	20.5	1105
16	Borneol	0.61	24.36	1183
17	Terpinene-4-ol	0.48	24.7	1190
18	*α*-Terpineol	0.08	25.49	1206
19	Carvacrol, methyl ether	0.04	27.38	1246
20	Thymol	19.51	29.61	1293
21	Carvacrol	65.22	30.57	1315
22	*E*-Caryophyllene	1.07	35.47	1427
23	Spathulenol	0.12	42.10	1590
24	Caryophyllene oxide	0.31	42.30	1595
	Other	0.08		
	Total	99.65		

**Table 2:** Effect of *Z*. *clinopodioides* essential oil, nisin, and their combination on *E*. *coli* O157:H7 in Doogh stored at 4°C.

Day	Control	Essential oil (%)		Nisin (IU/mL)		Essential oil (%) + nisin (IU/mL)			
		0.1	0.2	250	500	0.1 + 250	0.1 + 500	0.2 + 250	0.2 + 500
0	5.00 ± 0.00^aA^	5.00 ± 0.00^aA^	5.00 ± 0.00^aA^	5.00 ± 0.00^aA^	5.00 ± 0.00^aA^	5.00 ± 0.00^aA^	5.00 ± 0.00^aA^	5.00 ± 0.00^aA^	5.00 ± 0.00^aA^
1	4.84 ± 0.03^aA^	4.51 ± 0.08^bB^	4.22 ± 0.00^bcB^	4.87 ± 0.02^aA^	4.79 ± 0.00^aA^	4.16 ± 0.01^bcB^	4.38 ± 0.00^bcB^	3.98 ± 0.00^cB^	3.65 ± 0.02^dB^
3	3.72 ± 0.01^aB^	3.55 ± 0.03^bC^	3.45 ± 0.01^bC^	3.70 ± 0.01^aB^	3.68 ± 0.03^aB^	3.20 ± 0.00^bC^	3.55 ± 0.06^bC^	2.41 ± 0.01^cC^	2.11 ± 0.01^dC^
5	2.15 ± 0.02^aC^	ND	ND	2.10 ± 0.00^aC^	2.09 ± 0.01^aC^	ND	ND	ND	ND
7	ND	ND	ND	ND	ND	ND	ND	ND	ND
9	ND	ND	ND	ND	ND	ND	ND	ND	ND

^a^Means with different lowercase letters in the same row are significantly different (*p* < 0.05).

^A^Means with different capital letters in the same column are significantly different (*p* < 0.05).

ND: not detected.

**Table 3:** Acceptability scores (odour and flavour) of Doogh samples for different treatments.

Day	Control	Essential oil (%)		Nisin (IU/mL)		Essential oil (%) + nisin (IU/mL)			
		0.1	0.2	250	500	0.1 + 250	0.1 + 500	0.2 + 250	0.2 + 500
0	10 ± 0.0^a^	10 ± 0.0^a^	9.5 ± 0.0^a^	10 ± 0.0^a^	10 ± 0.0^a^	10 ± 0.0^a^	10 ± 0.0^a^	9.4 ± 0.0^a^	9.5 ± 0.0^a^
1	10 ± 0.0^a^	10 ± 0.0^a^	9.5 ± 0.0^a^	10 ± 0.0^a^	10 ± 0.0^a^	10 ± 0.0^a^	10 ± 0.0^a^	9.5 ± 0.0^a^	9.5 ± 0.0^a^
3	7.0 ± 0.7^b^	9.0 ± 0.2^b^	8.6 ± 0.2^b^	9.0 ± 0.0^b^	9.0 ± 0.0^b^	9.0 ± 0.1^b^	9.2 ± 0.2^b^	8.6 ± 0.1^b^	8.5 ± 0.0^b^
5	5.1 ± 0.1^c^	8.1 ± 0.3^c^	6.9 ± 0.3^c^	8.1 ± 0.1^c^	8.0 ± 0.2^c^	8.0 ± 0.2^c^	8.1 ± 0.1^c^	6.9 ± 0.1^c^	7.0 ± 0.2^c^
7	4.5 ± 0.1^c^	7.1 ± 0.0^d^	6.7 ± 0.1^c^	7.3 ± 0.1^d^	7.1 ± 0.1^d^	7.1 ± 0.3^d^	7.2 ± 0.3^d^	6.5 ± 0.2^c^	6.5 ± 0.1^d^
9	3.3 ± 0.1^d^	6.9 ± 0.0^e^	5.0 ± 0.1^d^	6.9 ± 0.0^d^	6.9 ± 0.0^d^	6.7 ± 0.1^d^	6.8 ± 0.3^d^	5.1 ± 0.2^d^	5.2 ± 0.0^e^

Means with different lowercase letters in the same column are significantly different (*p* < 0.05).
